# Adolescent Finswimmers: Early Myocardial Adaptations in Different Swimming Styles

**DOI:** 10.3390/sports6030078

**Published:** 2018-08-10

**Authors:** Vasileios Stavrou, Konstantinos Tsarouhas, Eleni Karetsi, Panagiotis Michos, Zoe Daniil, Konstantinos I. Gourgoulianis

**Affiliations:** 1 Laboratory of Cardio-Pulmonary Testing, Department of Respiratory Medicine, University of Thessaly, 41110 Larissa, Greece; ekaretsi@med.uth.gr (E.K.); panmichos@yahoo.gr (P.M.); zdaniil@med.uth.gr (Z.D.); kgourg@med.uth.gr (K.I.G.); 2 School of Physical Education and Sports Science, University of Thessaly, Karyes, 42100 Trikala, Greece; 3 Cardiological Department, General University Hospital of Larissa, 41222 Larissa, Greece; ktsarouhas14@yahoo.gr

**Keywords:** monofin, bifin, adolescent, myocardial hypertrophy

## Abstract

Background: The purpose of our study was to investigate early differences in the adolescent female finswimmers’ echocardiography parameters, possibly associated with different swimming-style training and different training equipment (monofin (MF) versus bifin (BF)). Method: Forty-three female finswimmers participated in our study (age: 15.6 ± 2.1 years, body mass index: 20.4 ± 2.2 kg/m^2^, body surface area: 1.56 ± 0.04 m^2^, body fat: 11.2 ± 0.6%) and were divided into two groups, according to the swimming style practiced (MF vs BF). Anthropometric characteristics, echocardiography and arterial pressure were measured. The independent *t*-test was used for statistical comparisons between groups. Stepwise multivariate regression analysis was applied to investigate associations between various variables. Results: The two groups used training equipment with different weights (*p* < 0.001). Female adolescent finswimmers presented signs of myocardial hypertrophy depicted by the increased left ventricle myocardial mass indexed to body surface area (101.34 ± 23.65). Different patterns of myocardial hypertrophy were observed for the two groups; MF swimmers presented concentric hypertrophy, while BF swimmers presented eccentric hypertrophy (relative wall thickness MF = 0.46 ± 0.08 vs BF = 0.39 ± 0.06 cm, *p* < 0.05). MF swimmers had also higher left ventricular posterior wall diameters (*p* < 0.05), lower stroke volume values (*p* < 0.05) and lower ejection fraction (*p* < 0.05) compared to BF athletes. Conclusion: Adolescent female finswimmers presented different patterns of myocardial hypertrophy possibly related to different training protocols and modes of exercise.

## 1. Introduction

Finswimming is a speed competition sport practiced at the surface or underwater with different monofins (MFs) or bifins (BFs) of variable rigidity. In MF, swimmers’ lower limbs are used for propulsion purposes (vertical displacement of the body) and in BF swimming athletes crawl with snorkel and fins.

Echocardiography is a noninvasive technique to evaluate the cardiac adaptations associated with training in people practicing sports [1]. According to Grazioli et al. [1], the hemodynamic changes caused by physical exercise alter the loading conditions of the heart and predispose one to cardiac adaptations in order to normalize the said loading stress. Myocardial wall thickness, stroke volume and both left ventricular (LV) and right ventricular (RV) end-diastolic diameters were greater in long-distance swimmers compared to short-distance swimmers. Moreover, the short-distance swimmers presented higher heart rate and systolic blood pressure values at rest compared to long-distance swimmers [2]. Cardiovascular adaptations are related to type and frequency of exercise and the kind of sport practiced [3]. Exercise duration relates to the morphological changes of the myocardial left ventricle (LV) including an increase in wall thickness [4], and usually affects both the interventricular septum (IVS) and left ventricular posterior wall (LVPW) [5]. LV wall thickness increase was observed in swimmers after aerobic exercise without any significant change in LV diastolic diameter [3]. Furthermore, regarding the physiology of finswimming, during training, periods of apnea may be used to cover short distances underwater, a practice that could be characterized as dynamic apnea, a phenomenon thatin turn could induce specific responses, that is, hypoxia and hypercapnia [6].

**Figure 1:**
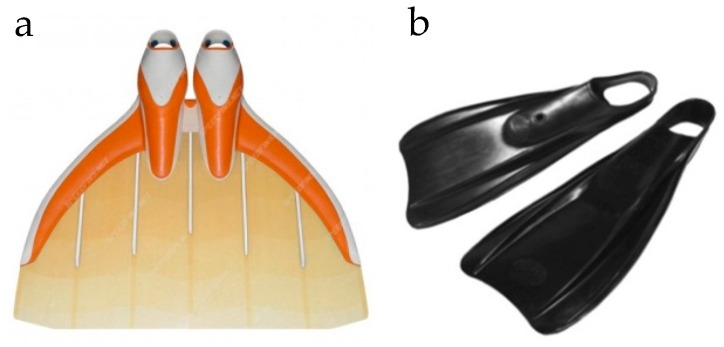
Athletes’ equipment: (**a**) monofin and (**b**) bifin. Monofin was made from rubber and fiberglass, and maximum weight is approximately 3.5 kg. Bifinswere made from rubber, and maximum weight is approximately 1.1 kg.

The purpose of the current study was to investigate the effect of swimming style and possibly the different training of the two adolescent groups on echocardiography parameters, and to evaluate early signs of myocardial adaptation to exercise. The two groups used different equipment (monofin (MF): fin with approximate weight 3.5 kg^−1^; and bifins (BFs): two fins with approximate weight 1.1 kg^−1^ (Figure 1)).

## 2. Method

### 2.1. Participants

Forty-three Caucasian young women from Greek finswimming sports clubs were included in the study on a voluntary basis (Table 1) and divided into two groups (MF_group_: *n* = 26 vs BF_group_: *n* = 17). Inclusion criteria include athletes involved in 50, 100 and 200 m events with MF on the surface and BF, training for at least 3 years, 10-h training per week and no medical history. All participants trained after school between 14:30 to 16:30, 5 to 6 days per week, and used equipment in accordance with the World Underwater Federation rules (CMAS) [7]. Swimmers with medical history, as well as swimmers involved in classical swimming training and/or other sports for more than one year, were excluded from the study population. The study period was 1 month. The study was conducted according to the Helsinki declaration for use in human subjects (No. of Ethical Committee: 2-5/2.2.2011, University of Thessaly). All participants’ parents submitted a written consent.

### 2.2. Data Collection

Anthropometric characteristics as body height, body mass (Seca 700), body mass index (BMI = kg/m^2^), body surface area (BSA = (height _(cm)_ × weight _(kg)_)/3600 ½) [8], percentage of body fat (7-point measurement, Harpenden),stages of biological maturation [9] and pulmonary function parameters (FEV_1_: forced expiratory volume in 1st second, FVC: forced vital capacity, PEF: peak expiratory force; MasterScreen-CPX, VIASYS HealthCare, Hochberg, Germany) [10] were recorded prior to echocardiography.

Echocardiographic studies were performed in the morning hours and all participants did not compete or train for at least 12 h before the study. A certified cardiologist performed the echocardiography examinations and analyzed the echocardiographic findings. A second certified cardiologist blinded to the athletes reviewed the results. Two-dimensional echocardiography was performed, with the subjects resting in a left lateral decubitus position, using a Vivid BT08 (General Electric, Miami, FL, USA). Heart images were obtained in the standard parasternal long-axis and short-axis and apical four-and-two chamber planes. Left ventricle wall thickness (LVWT) was measured from 2D short-axis views at enddiastole, with the greatest measurement within the left ventricular (LV) wall defined as the maximal wall thickness. M-mode echocardiograms derived from 2D images in the parasternal long axis were used for the measurement of LV end-diastolic and systolic dimensions according to EAE/ASE [11] and the American Society of Echocardiography [12]. The biplane method of disks (modified Simpson’s rule) was used to assess left ventricle ejection fraction and volumes. Left ventricle mass was calculated with the use of the linear method (Equation (1)):

LV mass (g) = 0.8(1.04(((LVEDD + IVSd + PWd)^3^ − LVEDD^3^))) + 0.6g. (1)

Relative wall thickness (RWT) was calculated with the use of the formula RWT = 2 × (posterior wall thickness)/(LV internal diameter at end diastole), and consequently, any observed increase in LV mass was categorized as either concentric (RWT > 0.42) or eccentric (RWT ≤ 0.42) hypertrophy [12]. Arterial pressure was measured using manual cuff manometry (Mac, Oita, Japan).

### 2.3. Data Analyses

The Kolmogorov-Smirnov test was used to evaluate the normality of the distribution. The independent *t*-test was used for statistical comparisons between groups. Stepwise multivariate regression analysis was used to investigate associations between various variables. The level of significance was set to *p* < 0.05 and the data are presented as mean value and standard deviation (mean ± SD). The SPSS 15 statistical package (SPSS Inc., Chicago, IL, USA) was used for the statistical analyses. Study power calculations were made with the use of Java Applets for Power and Sample Size [13].

## 3. Results

**Figure 2:**
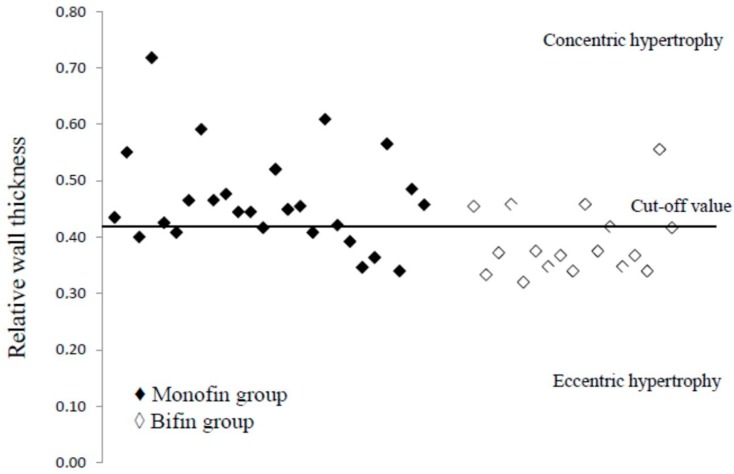
Relative myocardial wall thickness between groups. Cut-off value of concentricvs eccentric hypertrophy is considered RWT = 0.42.

The two groups used training equipment with different weights (MF: 3.5 ± 0.6 vs BF: 1.1 ± 0.4 kg^−1^, *p* < 0.001). Female adolescent finswimmers presented signs of myocardial hypertrophy evident by the increased left ventricle myocardial mass indexed to body surface area (101.34 ± 23.65 g/m^2^), with different patterns of myocardial hypertrophy for the two groups. MF swimmers presented concentric hypertrophy while BF swimmers presented eccentric hypertrophy (relative wall thickness MF = 0.46 ± 0.08 vs BF = 0.39 ± 0.06, *p* < 0.05, Figure 2).

MF swimmers had also higher left ventricular posterior wall diameters (LVPW; MF: 1.03 ± 0.17 vs. BF: 0.91 ± 0.09 cm, *p* < 0.05), lower stroke volume values (MF: 56.6 ± 16.2 vs BF: 70.7 ± 14.9 mL, *p* < 0.05) and lower values in ejection fraction (MF: 59.5 ± 10.2% vs BF: 67.8 ± 5.0%, *p* < 0.05) compared to BF athletes. Pulmonary function tests found that the two groups had comparable FVC and PEF but differed in FEV_1_ with the BF group presenting higher values (MF: 112.8 ± 5.9% vs BF: 120.9 ± 6.9% of predicted values, *p* < 0.001).

Multivariate stepwise regression analysis found that LV myocardial hypertrophy and specifically LV mass indexed to BSA was weakly (R = 0.42 for the model) associated only with swimmers’ age (beta = 0.365, *p* < 0.05) and not significantly with pool training hours per week (beta = 0.255, *p* = 0.085). At the same time, multivariate stepwise regression analysis showed that RWT was associated (R = 0.59 for the model) with age (beta = −0.479, *p* < 0.05), training hours spent at the gym (beta = 0.412, *p* < 0.05), and PEF measured by spirometry (beta = 0.395, *p* < 0.05), but not significantly with BMI (*p* > 0.05) and not significantly with swimmers’ height (*p* > 0.05).

The results of athletes’ characteristics, training frequency and performance are presented in Table 1. Hemodynamic parameters, left ventricle volumes and function, cardiac dimensions and left ventricle myocardial mass are presented in Table 2.

## 4. Discussion

Cardiac adaptation to exercise training encompasses morphological, functional and electrical changes that are referred to as the “athletic or athletes’ heart”. Acute endurance exercise represents a significant stress to the heart. According to Morganroth [14], an eccentric LV hypertrophy is observed in endurance athletes evident by increased LV internal dimension and mass, with minor changes in LV wall thickness. Heart pump efficiency depends on the morphological characteristics and size of heart cavities along with the influence in cardiac function of the blood volume that the ventricles are receiving in the diastole, and the resistance the vessel walls raise during blood extrusion [15].

Female adolescent finswimmers of the present study were characterized by different patterns of myocardial hypertrophy based on the investigated variants of the said sport. It can be assumed that myocardial adaptation to exercise was the culprit of the myocardial hypertrophy and that the origin of the different adaptations could be derived from the different physiological demands when swimming with BF or MF. Higher SV and LVEF values observed in the BF group could be attributed to the aerobic nature of exercise that BF swimming style can be regarded as, where more muscle groups are involved. At the same time, MF swimming demands a body position with high tension from the waist up, which probably augments vascular resistance during exercise and imposes an increase (afterload) to swimmers’ myocardium, leading to concentric hypertrophy. In finswimming and especially in athletes who use the monofin, the thighs have more activation during swimming compared to other muscle groups [16]. Isometric and isotonic exercises are components of the exercise physiology and usually do not occur in isolation, as most sport activities involve a combination of these two principal forms of hemodynamic stress [17]. As such, MF swimming probably is associated with high loads of isometric stress, along with isotonic stress.

Previous studies demonstrated that changes in left ventricular mass and ventricular cavity size with physical training and conditioning take place simultaneously with augmented oxygen uptake, suggesting that ventricular hypertrophy is associated with better cardiopulmonary measures [18]. Exercise-associated overload represents the primary mechanism responsible for cardiac structure changes [18]. These adjustments are related to body mass index, body surface area and the frequency of exercise [18]. Systematic physical training tests the ability of the cardiovascular system to supply oxygen to the muscles under exercise stress. Left ventricular hypertrophy is regarded as compensatory or adaptive to a hemodynamic stimulus, representing pressure and/or volume overload, and is related to the type of physical exercise, duration and intensity of the exercise [19], and different equipment such as those used by the athletes of our study.

Results from our study reveal differences between groups because of equipment. During the training but not the race, finswimmers’ periods of apnea may be used to cover short distances underwater. This practice, which is characterized as dynamic apnea, is beneficial for performance in sports [20].

Previous study reveals that different breathing techniques during finswimming can affect differently the heart rate, arterial oxygen saturation and the maximal inspiratory pressure [6]. The said differences are explained by the different intrathoracic pressure developed during the swimming trails, as well as the systematic changes in the breathing pattern, which are necessary in order to optimize muscle strength and increase endurance [6]. Moreover, it has been proposed that the physiological characteristics of finswimming athletes [21] and cardiac adaptation to exercise training depend on the different muscle groups activated during the workout [22], while the cardiac remodeling in athletes is more pronounced in the heart cavities with specific regional differences [23]. During the training and the race, finswimmers develop periods of apnea that may be used to cover short distances underwater. This practice, which is known as dynamic apnea, is thought to be beneficial for performance enhancement [20]. It is found that Valsalva physiology at the time of isometric activity, which also stands in prolonged breath holding during finswimming, can counteract left ventricular pressure overload because of the simultaneous increases in intrathoracic and intracardiac pressure, and keep the myocardial transmural pressure, which determines the myocardial work, in a normal range [24,25].

The mechanisms underlying strength improvements in prepubertals arise through an interaction between neural and hormonal mechanisms, and affect the cardiovascular system, during endurance training in adult athletes, with major changes in morphological and functional adaptations of the heart. In the present study, myocardial hypertrophy of female finswimmers was evident compared to published data on relevant age groups with the use of magnetic resonance imaging [26]. In an earlier study with the use of MRI, Cain et al. found that female adolescents presented LVM/BSA = 71 ± 10 g/m^2^ [27].

The findings of the current study could also permit clinicians to accurately diagnose and treat BF swimmers presenting themselves as patients, since the observed concentric hypertrophy is part of the adaptive physiology and not a pathological condition. The overlap between physiology and pathology is important in clinical medicine and especially in cases of myocardial hypertrophy in athletes where highly trained athletes present themselves to primary care providers for clearance to participate in sports or with the question of returning to training after minor events or symptoms [24].

## 5. Limitations of Our Study

A limitation of the present study is that the adolescent female finswimmers studied were all Caucasians, meaning that the results may not apply to other ethnic groups. Moreover, athletes were evaluated only by echocardiography and not with the use of magnetic resonance imaging. Finswimmers’ myocardial right ventricle function was not investigated in the current study and LV function was only investigated with “classical parameters”.

## 6. Conclusions

Adolescent female finswimmers presented different patterns of myocardial hypertrophy possibly related to different training programs and modes of exercise. MF swimming induced concentric cardiac hypertrophy, while BF induced eccentric hypertrophy. The said myocardial adaptations to exercise could be attributed to the different physiological demands imposed by the two swimming styles.

## Figures and Tables

**Table 1:** Athletes’characteristics, training frequency and performance. Continuous variables are presented as mean ± standard deviation.

Characteristics	Total (*n* = 43)	MF_group_ (*n* = 26)	BF_group_ (*n* = 17)	*p*-Value
Age, years	15.6 ± 2.1	15.9 ± 2.1	15.1 ± 2.1	NS
Body mass index, kg/m^2^	20.4 ± 2.2	20.7 ± 2.2	19.8 ± 2.1	NS
Body surface area, m^2^	1.5 ± 0.04	1.6 ± 0.04	1.5 ± 0.03	NS
Body fat, %	11.2 ± 0.6	11.4 ± 0.6	10.9 ± 0.7	NS
Tanner score	4.1 ± 0.7	4.0 ± 0.7	4.3 ± 0.8	NS
Training age, years	4.1 ± 1.5	4.5 ± 1.8	3.4 ± 0.5	0.017
Training, day/h	2.1 ± 0.2	2.1 ± 0.2	2.0 ± 0.1	NS
Training, week/h	13.0 ± 1.0	13.1 ± 3.1	13.2 ± 2.9	NS
Gym, week/h	3.0 ± 1.1	3.2 ± 0.9	2.8 ± 1.3	NS
Best trial				
50-m, second	23.4 ± 2.6	22.0 ± 1.8	26.5 ± 1.1	<0.001
100-m, second	55.7 ± 5.7	51.5 ± 2.4	59.4 ± 5.1	0.003
200-m, second	113.4 ± 10.1	106.6 ± 4.5	124.6 ± 3.6	0.001

*Note*: NS: not significant.

**Table 2:** Hemodynamic, left ventricular volume and function, intracardiac dimension parameters, left ventricle myocardial mass and respiratory parameters between groups. Continuous variables are presented as mean ± standard deviation.

Hemodynamic, LV and Respiratory Parameters	Total (*n* = 43)	MF_group_ (*n* = 26)	BF_group_ (*n* = 17)	*p*-Value
Hemodynamic				
HR_resting_, bpm^−1^	78.8 ± 9.2	80.1 ± 7.8	76.9 ± 10.9	NS
SBP_resting_, mmHg	111.5 ± 8.4	113.4 ± 8.8	108.4 ± 7.1	NS
DBP_resting_, mmHg	71.4 ± 4.6	72.1 ± 4.7	70.2 ± 4.1	NS
Left ventricular volume and function				
EDV, mL	101.2 ± 20.9	97.3 ± 18.7	107.3 ± 23.1	NS
ESV, mL	38.1 ± 12.9	41.0 ± 14.5	33.6 ± 8.4	0.040
SV, mL	62.2 ± 17.1	56.6 ± 16.2	70.7 ± 14.9	0.006
EF, %	62.8 ± 9.4	59.5 ± 10.2	67.8 ± 5.0	0.001
Intracardiac dimensions				
IVSd, cm	1.0 ± 0.2	1.0 ± 0.2	1.0 ± 0.1	NS
IVSd indexed, cm/m^2^	0.63 ± 0.1	0.63 ± 0.1	0.62 ± 0.1	NS
LVIDd, cm	4.5 ± 0.8	4.3 ± 0.9	4.7 ± 0.4	NS
LVIDd indexed, cm/m^2^	2.94 ± 0.3	2.87 ± 0.3	3.06 ± 0.3	NS
LVPWd, cm	0.99 ± 0.2	1.0 ± 0.2	0.9 ± 0.1	0.015
LVPWd, cm/m^2^	0.63 ± 0.1	0.66 ± 0.1	0.59 ± 0.1	0.03
Left ventricle myocardial mass				
LV mass, g	158.3 ± 37.7	160 ± 39.9	155.4 ± 34.9	NS
LV mass indexed, g/m^2^	101.3 ± 23.6	102 ± 24.8	100.3 ± 22.5	NS
Pulmonary function tests				
FEV_1_, %pred.	116 ± 7.5	112.8 ± 5.9	120.9 ± 6.9	<0.001
FVC, %pred.	114.9 ± 7.9	114 ± 7.7	116 ± 8.3	NS
PEF, %pred.	106.7 ± 5.4	107.7 ± 6.2	105 ± 3.5	NS

*Note:* DBP: diastolic blood pressure, EDV: end-diastolic volume, EF: ejection fraction, ESV: end-systolic volume, FEV_1_: forced expiratory volume in 1s, FVC: forced vital capacity, HR: heart rate, IVS: interventricular septum thickness, LV mass indexed: left ventricle myocardial mass indexed to body surface area (BSA), LV mass: left ventricle myocardial mass, LV: left ventricular, LVID: left ventricular internal dimension, LVPW: left ventricular posterior wall, NS: not significant, PEF: peak expiratory force, SBP: systolic blood pressure, SV: stroke volume.
